# Our love-hate relationship with DNA barcodes, the Y2K problem, and the search for next generation barcodes

**DOI:** 10.3934/genet.2018.1.1

**Published:** 2018-01-17

**Authors:** Jeffrey M. Marcus

**Affiliations:** Department of Biological Sciences, University of Manitoba, Winnipeg, MB, Canada, R3T 2N2

**Keywords:** DNA barcoding, year 2000 problem, millennium bug, mitochondrial genome evolution, Trichoptera, Genome skimming, Next Generation Barcodes, *Cheumatopsyche*, *Hydropsyche*, *Potamyia*

## Abstract

DNA barcodes are very useful for species identification especially when identification by traditional morphological characters is difficult. However, the short mitochondrial and chloroplast barcodes currently in use often fail to distinguish between closely related species, are prone to lateral transfer, and provide inadequate phylogenetic resolution, particularly at deeper nodes. The deficiencies of short barcode identifiers are similar to the deficiencies of the short year identifiers that caused the Y2K problem in computer science. The resolution of the Y2K problem was to increase the size of the year identifiers. The performance of conventional mitochondrial *COI* barcodes for phylogenetics was compared with the performance of complete mitochondrial genomes and nuclear ribosomal RNA repeats obtained by genome skimming for a set of caddisfly taxa (Insect Order Trichoptera). The analysis focused on Trichoptera Family Hydropsychidae, the net-spinning caddisflies, which demonstrates many of the frustrating limitations of current barcodes. To conduct phylogenetic comparisons, complete mitochondrial genomes (15 kb each) and nuclear ribosomal repeats (9 kb each) from six caddisfly species were sequenced, assembled, and are reported for the first time. These sequences were analyzed in comparison with eight previously published trichopteran mitochondrial genomes and two triochopteran *rRNA* repeats, plus outgroup sequences from sister clade Lepidoptera (butterflies and moths). *COI* trees were not well-resolved, had low bootstrap support, and differed in topology from prior phylogenetic analyses of the Trichoptera. Phylogenetic trees based on mitochondrial genomes or *rRNA* repeats were well-resolved with high bootstrap support and were largely congruent with each other. Because they are easily sequenced by genome skimming, provide robust phylogenetic resolution at various phylogenetic depths, can better distinguish between closely related species, and (in the case of mitochondrial genomes), are backwards compatible with existing mitochondrial barcodes, it is proposed that mitochondrial genomes and *rRNA* repeats be used as next generation DNA barcodes.

## 1. Introduction

### 1.1. DNA barcoding

DNA barcodes were initially proposed as a solution to the worldwide shortage of taxonomic expertise for many groups of organisms [1]. Short stretches of sequenced DNA from a single gene from expertly diagnosed specimens from as many species as possible would serve as a database of identifiers or barcodes to facilitate the identification of additional unknown specimens collected in the future. For animals, a piece of the mitochondrial *cytochrome c oxidase subunit I* (*COI*) gene (Table 1) was employed as the barcode sequence because of the availability of degenerate PCR primers that had been shown to consistently amplify a homologous DNA fragment in diverse organisms [2]. In fungi, a fragment of the *internal transcribed spacer 2* (*ITS2*) within the nuclear *ribosomal RNA* (*rRNA*) repeat was adopted as the barcode region [3]. In bacteria, variable portions of the *16S rRNA* are used for barcoding [4],[5] In plants, regions of 2 different chloroplast genes are used as a combinatorial barcode: the large subunit of *ribulose bisphosphate carboxylase* (*rbcL*) and *maturaseK* (*matK*) [6]. The choice and size of each of these barcode regions was based on the technologies for PCR amplification and DNA sequencing that were widespread when the barcode primers were designed [7]. DNA barcoding in animals has been the most controversial as discussed below and will be the focus of my comments here, but many of the challenges confronting DNA barcoding in animals also confront the barcoding strategies employed in other taxa. The suggested approach to overcoming these challenges should be broadly applicable to many groups of organisms.

Mitochondrial *COI-*based DNA barcodes are often incredibly helpful for associating morphologically distinct life stages or sexes within a species, identifying cryptic species, and understanding the diversity of species assemblages within a particular habitat or geographic region [21]–[25]. The strengths of barcoding include the standardization of the identifiers across taxa (at least within Kingdoms—different barcode loci are used in animals, plants, fungi, and bacteria (Table 1)), the large number of species that have already been barcoded, and the fact that many different laboratories can contribute to a unified barcoding database using a variety of different experimental approaches and instrumentation [12],[26]–[30]. Because they are useful in many different contexts, a great deal of investment has been made in expanding the number of species and populations that have been DNA barcoded, as well as in computational resources for using the database of barcodes [12]. It is because of this utility that they have been so enthusiastically embraced by so many researchers [17],[31]–[33].

**Table 1.** Commonalities between challenges presented by the Y2K problem and by DNA barcoding.

	**Y2K Problem**	**DNA Barcoding**
	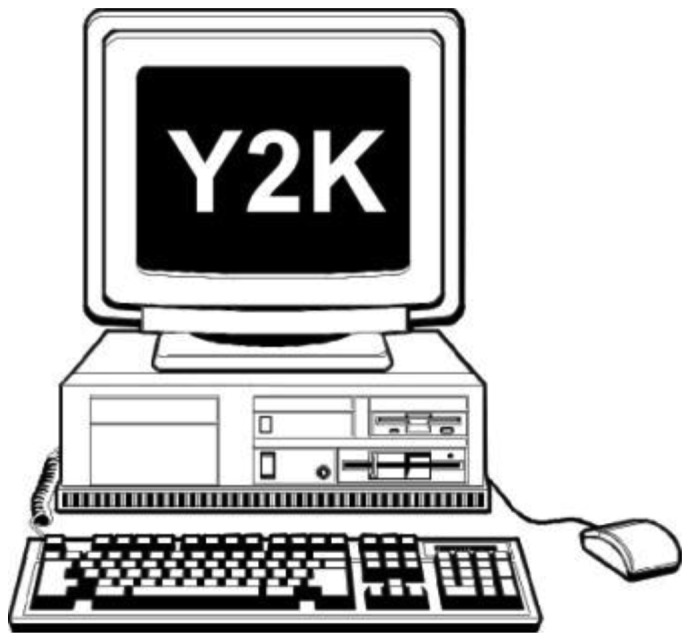	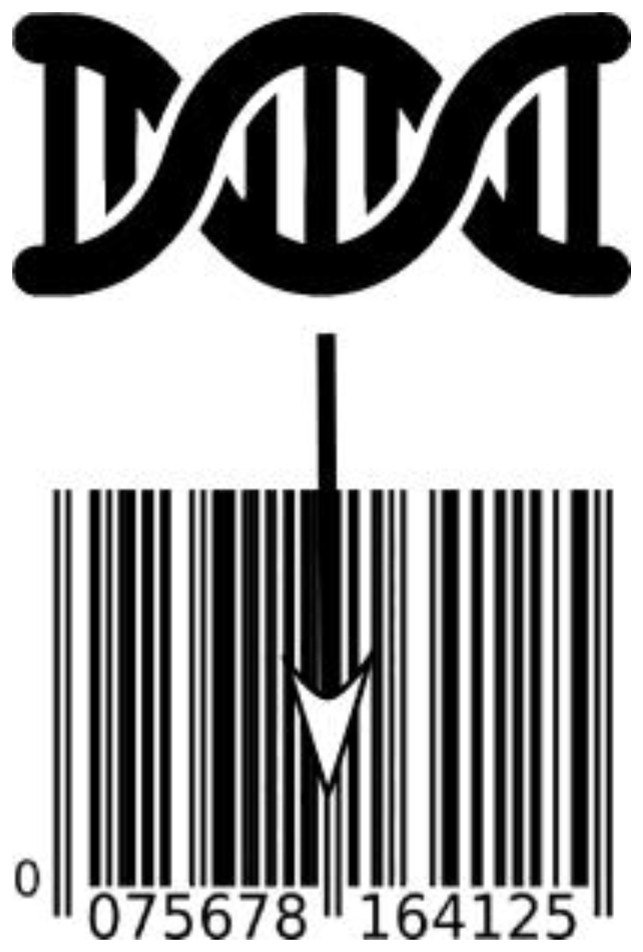
Identifier	two-digit year notation	animals: ∼658 bp *COI* fragment plants:∼553 bp *rbcL* & ∼776 bp *matK* fragments fungi:∼534 bp *ITS2* fragment bacteria:∼1400 bp *16S rRNA* fragment
Identifier purpose	1. To distinguish between years 2. To sort data chronologically 3. Mathematical operations (mostly addition/subtraction) to calculate time intervals [8]	1. To facilitate species identification without reference to morphology 2. To determine species limits and potentially to detect new species (by % divergence) 3. To understand species relationships [1],[9]
Constraints influencing the original design of the identifier	Extremely limited memory in early computers (No longer applicable by 1983 when hard drives became common in personal computers [10])	1. ∼500 bp maximum read length of radiolabeled dideoxy-terminated sequencing (No longer applicable by 1996 when automated dye-labeled sequencing radiolabeled sequencing [7]) 2. Need for widely-conserved primer binding sites for PCR amplification from diverse organisms (No longer applicable by 2007 when next generation sequencing methods were introduced which could recover high copy number genes without PCR [11])
Reason(s) for identifier maintenance	To maintain backwards compatibility with older software applications Reuse of old computer algorithms in newer code Inertia/tradition/habit [10]	To take advantage of the large database of existing DNA barcodes To facilitate comparisons of new results with those of previous studies [12] Cost Inertia/tradition/habit [13]
Crisis	At the turn of the 21st century: 2000 > 1999, but 00 < 99. Compromised sorting algorithms and mathematical operations [10]	1. Many recent species pairs cannot be separated by 658 bp barcodes because there has not been enough time for mutations to accumulate within the regions [14]. 2. Barcodes from organelle genomes in plants and animals are vulnerable to lateral transfer between species (through hybridization or other mechanisms) and reticulate evolution, sometimes resulting in misidentification [15],[16]. 3. The barcode region reaches saturation quickly and cannot resolve deep phylogenetic nodes. (e.g., amphibians saturate at 10–11%, reptiles saturate at 9–10%, holometabolous insects saturate at 22%, and all hexapods saturate at 25% barcode sequence divergence) [17]. Collectively these issues compromise the general utility of DNA barcode application. It is highly desirable to produce more universal DNA barcodes that address these deficiencies [18]
Resolution	Enlarge Identifiers: Worldwide effort to update software applications and change to four-digit identifiers in the late 1990s (acceptable until the year 9999) [10],[19]	Enlarge and diversify identifiers: The high copy number of organelle genomes and the nuclear rRNA repeat relative to the rest of the nuclear genome will cause these sequences to be very well represented among random reads of whole genome DNA extractions [20]. This permits routine assembly of complete organelle genomes (e.g., mitochondrial genome, ∼15 kb) and complete rRNA repeat sequences (∼9 kb). These longer sequences contain segments that evolve at different rates and have much higher information content than the short barcode sequences currently in use. Here I explore the use of these sequences as next generation barcodes to address the deficiencies of DNA barcodes currently in use.

However, other researchers have pointed out that while DNA barcoding uses the phylogenetic species concept (defining species as reciprocally monophyletic clades, with each clade possessing a distinct set of diagnostic characteristics [34]), its implementation often ran contrary to some of the guiding principles of phylogenetics [13],[35],[36]. For example, the tree-building algorithms implemented in the Barcode of Life Database (BOLD) are distance-based and do not use rigorous phylogenetic approaches to understanding relationships among barcodes [37],[38]. Also, by relying exclusively on sequences from organelle genomes, DNA barcoding activities in animals and plants are vulnerable to misleading associations between species and DNA barcodes because of the frequency of interspecific organelle capture due to hybridization or other kinds of lateral transfer events (Table 1) [16],[39]–[42]. Finally, phylogenetic hypotheses (including the identification of monophyletic groups of barcodes associated with individual species) based on a small number of informative characters are prone to unresolved [43] or erroneous relationships [18],[44] between branches, as well as low bootstrap support [45]. This manifests both in recently diverged species that have not yet accumulated sequence variation within the barcode region [29] and in more distantly related species where multiple substitutions at the same sites obscure the phylogenetic signal within barcodes [17]. To the extent that any taxon defies the phylogenetic species concept's fundamental criterion of reciprocal monophyly (due to lack of sequence divergence between species, retained ancestral polymorphisms, organelle capture, parallel evolution, etc.) the conventional DNA barcoding approach will fail, even for its original intended purpose of identifying species.

The question is whether it is possible to build upon the strengths of the DNA barcoding strategies currently in use, while also addressing these deficiencies. In this work, I will suggest that the Y2K problem from computer science [10] shares a number of common features with DNA barcoding, and so the solutions to the challenges of DNA barcoding in its current implementation may also share some similarities to how the Y2K problem was resolved.

### 1.2. The Y2K problem

The Y2K problem in computer sciences (Table 1) refers a feature of many computer programs in the late 1990s that encoded year identifiers with only two digits (for example: such that the year “1997” would be identified by “97”) [8]. The practice of using two-digit identifiers dates to the early days of computer science when computer memory was at a premium, and so the minimum number of digits necessary to encode year identifiers was employed. In turn, the adequacy of minimal two-digit year identifiers was due to the historical contingency of the birth of computer science in the 1940s and 1950s, decades away from both the turn of the 20^th^ century, and the turn of the 21^st^ century [10].

The practice of using two-digit identifiers was perpetuated over decades, long after the constraints of computer memory no longer applied, as code from earlier applications was reused in subsequent applications, perhaps abetted by habits and traditions adopted by programmers over time [10]. For much of the 20^th^ Century, this practice was unproblematic, but as the year 2000 approached it became apparent that this usage would be problematic for distinguishing between years from different centuries (1901 vs 2001), for sorting data chronologically (01 < 99, but 2001 > 1999), and for mathematical operations (01 − 99 = −98, but 2001 − 1999 = 2). The solution to the Y2K problem in the late 1990s, was a coordinated worldwide effort to update computer software so that they employed four-digit year-identifiers [10],[19]. The choice of four-digit identifiers was arbitrary, but should allow for upgraded computer software to function as expected until the year 9999.

The sizes of the DNA barcodes now in use are similarly arbitrary. They were selected on the basis of several factors including the availability of conserved primers that would amplify the barcode region in diverse organisms, and the availability of a large collection of previously sequenced examples of the region from many species [1]. Both of these factors were greatly influenced by the early experimental methods being used to acquire barcode sequences. The *COI* primers that came to be the standard for animal barcodes were described in 1994 [2] when most DNA sequencing was done by ^32^P-labelled dideoxy-terminated Sanger sequencing, which has a maximum read length of about 500 bp [7]. By sequencing in both directions, all of the 658 bp *COI* fragment could be covered, with bidirectional coverage over most of the more variable interval between the two more conservative binding sites. Radiolabelled Sanger sequencing was largely replaced by fluorescent dye-labeled Sanger sequencing, by about 1996, when the cost of the dye-labeled technology dropped below the cost of radio-labeled sequencing [7]. Yet, the same 658 bp region continued to be extremely popular for phylogenetic studies [46],[47], and for DNA barcoding initiatives (which were introduced in 2003) [1], even though fluorescent dye-labeled Sanger sequencing has much longer maximum read lengths.

Beginning in 2007, when next generation sequencing technologies began to be adopted by the research community [11], it became possible to recover high copy number sequences like the mitochondrial genome (including the *COI* barcode region) by low-coverage shotgun sequencing of the whole genome or “genome skimming” [48]–[51] without requiring the use of conserved PCR primers flanking the barcode region. Similarly, the chloroplast genome of plants and the nuclear *rRNA* repeat (which contains the *18S*, *5.8S*, and *28S rRNAs* and *internal transcribed spacer* (*ITS)* 1 and 2 sequences) that also occur at high copy number are also easily recovered by genome skimming [49]. It has already been demonstrated that these high copy number sequences contain substantial phylogenetic signal [50],[51]. Complete mitochondrial genome sequences in particular have a good track record for reconstructing the phylogenetic history of organisms at a range of taxonomic (and sequence) divergence [52]–[56].

While it is still more affordable to use traditional DNA barcodes, but this may always not be the case. In 2018, it costs roughly $10 USD to sequence a 658 bp *COI* barcode PCR product in both directions by dye-terminated Sanger sequencing. In comparison, to sequence a complete mitochondrial genome and a complete nuclear rRNA repeat by genome skimming using an Illumina MiSeq instrument as described in this paper costs approximately $250 USD per sample, while yielding a vastly larger pool of sequence data for analysis. At the same time, while the cost of Sanger sequencing has been fairly stable for the last 15 years (2002–2017), the cost of next generation sequences dropped from $0.08 USD per raw Megabase of DNA sequence to less than $0.02 USD per raw Megabase between 2014 and 2017 [57]. It is therefore becoming increasingly affordable to generate next generation sequence datasets for the purposes of species identification and phylogenetics.

Once a system of identifiers exists, it is often used for purposes not envisioned when it was first created. A fundamental element of the analogy between the Y2K problem and DNA barcoding is that in both cases, many of the fundamental limitations of the identifiers were not apparent until practitioners attempted to extend their use to novel situations. In the case of two-digit year identifiers, this was when then number of years being identified exceeded 100; while in the case of DNA barcodes, it was when researchers tried to use DNA barcode identifiers for molecular phylogenetic and population genetic analysis beyond mere species identification.

It is to address two issues that I am arguing that a major modification of the current DNA barcoding strategy may be warranted. First, that DNA barcodes as currently implemented are imperfect tools even for their original intended purpose of species identification. Second, that the current DNA barcodes are inadequate or inappropriate for many of the applications for which many researchers wish to use molecular species identifiers. As an explicit analogue to the decision to expand the number of digits in year identifiers in order to resolve the Y2K problem, this is an opportune time to see if easily (and increasingly inexpensively) obtained plastid genome and nuclear ribosomal repeat sequences might be used as larger “next generation” DNA barcodes that might be less vulnerable to some of the deficiencies of the short conventional barcode sequences that are currently in use [18]. That is not to say that the current DNA barcodes have no role to play (my research group uses them frequently in our own work [44],[58]–[60]), but rather that we may be able to design next generation barcodes that have all of the positive attributes of the current identifiers, while eliminating most of the limitations that have plagued DNA barcoding efforts to date.

### 1.3. The test case: the net-spinning caddisflies (Insecta: Trichoptera: Hydropsychidae)

For a test case to explore whether enlarging barcodes can improve their performance for both species identification and phylogenetic analysis, I chose to examine caddisflies (Insect Order Trichoptera) with a focus on Family Hydropsychidae, the net-spinning caddisflies. The species in the Hydropsychidae are distinctive because they spin nets made of silk that they use to harvest food particles from the water column in their larval aquatic environment [61]. The Trichopera demonstrate some of the most frustrating limitations of current barcodes: the family-level phylogeny of the Trichoptera cannot be reconstructed on the basis of barcode sequences [62] and within the Hydropsychidae, there are many similar species that can be difficult to distinguish on the basis of morphology (especially as larvae) and that also cannot always be distinguished on the basis of *COI* barcodes due the presence of shared haplotypes [12],[63].

The family-level phylogeny of the Trichoptera is well established on the basis of multiple datasets [64],[65], as is the sister clade relationship between the Trichoptera and the Lepidoptera (butterflies and moths) [66],[67], but the species-level phylogeny of many of the 14,500 described trichopteran species is unknown. It has recently been proposed that *COI* barcodes can and should be used to arrange the terminal branches of the caddisfly phylogeny, in combination with more extensive sequence data from other genetic regions from select species to establish the backbone and deeper nodes of the tree [65]. Yet, in some other taxonomic groups, barcode-based phylogenies are not good predictors of the phylogeny of the mitochondrial genomes of which they are a part [18],[44]. Assessing the predictive validity of *COI* barcode-based phylogenies in the Trichoptera would be very helpful to determine if the proposed strategy for resolving terminal branches [65] is likely to be successful. Therefore the explorations of conventional versus next generation barcode approaches considered here will yield valuable insights both for future phylogenetic research in the Trichoptera and for all taxa more generally.

To evaluate the effectiveness of these different approaches to barcoding, datasets of *COI* barcodes and mitochondrial genomes were assembled for 14 trichopteran species, and a dataset of *rRNA* repeats was assembled for 8 trichopteran species. All of the *rRNA* repeat sequences and all but 6 of the mitochondrial genome sequences were collected and assembled by my laboratory for this study. The data sets include 3 species of *Cheumatopsyche* (Hydropsychidae) that are not readily distinguishable by *COI* barcodes, 3 species of *Hydropsyche* (Hydropsychidae) that are distinguishable by *COI* barcodes, and 1 species of *Potamyia* (Hydropsychidae), as well as 7 caddisfly species from 6 other trichopteran families. Also included in the data sets were sequences from representatives of 2 lepidopteran families as outgroups. These analyses consider all of the publicly available complete mitochondrial genomes and *rRNA* repeats for the Trichoptera.

**Table 2.** Caddisfly species collected at the Living Prairie Museum and analyzed in this study.

			Million	Mitochondrial Genome			Nuclear *rRNA* Repeat		
Scientific Name	Collection Date	Specimen Identifier	Reads Total	# Reads	Mean Fold Coverage	Length (bp)	#Reads	Mean Fold Coverage	Length (bp)
Hydropsychidae									
*Cheumatopsyche analis^1^*	14-Aug-15	2015.08.14.065A	6.84	67275	1266 X	15097	9412	308 X	7791
*Cheumatopsyche campyla^1^*	17-Jul-15	2015.07.17.021A	5.89	67559	1275 X	15100	6239	122 X	8323
*Cheumatopsyche speciosa*	14-Aug-15	2015.08.14.106A	7.75	37191	184 X	15098	29449	548 X	8683
*Hydropsyche orris*	14-Aug-15	2015.08.14.066A	2.08	86392	458 X	15185	29054	640 X	9228
*Hydropsyche simulans*	14-Aug-15	2015.08.14.067	6.30	15864	326 X	15237	31093	301 X	7797
*Potamyia flava*	14-Aug-15	2015.08.14.070B	4.59	122730	600 X	15160	53095	1222 X	9244
Limnephilidae									
*Anabolia bimaculata*	17-Jul-15	2015.07.17.018	8.29	40865	482 X	15048	98766	3149 X	9400
Leptoceridae									
*Triaenodes tardus*	14-Aug-15	2015.08.14.077	8.36	6952	35 X	14963	82832	168 X	9232

^1^Read length for *C. analis* and *C. campyla* was 300 bp. For all other species, read length was 75 bp.

## 2. Materials and Methods

### 2.1. Specimen Collection and DNA Preparation

Adult caddisflies (Insecta: Trichoptera) were collected by USDA blacklight trap containing ethyl acetate [68] deployed overnight as part of a taxonomic inventory of arthropods at the Living Prairie Museum in Winnipeg, Manitoba, Canada (GPS 49.889607 N, −97.270487 W) during the 2015 growing season. The Living Prairie Museum consists of 12.9 hectares of relict unplowed prairie maintained by periodic controlled burns and is home to over 160 native plant species, supporting a rich arthropod fauna [69]. Nearby aquatic habitats suitable for larval caddisflies include Sturgeon Creek (0.57 km) and the Assiniboine River (1.92 km). Light trap collections were brought back to the laboratory, sorted to species by morphology, and then stored in glassine envelopes at −20 °C before further processing. Specimens collected as part of the inventory that are used in this study include 6 species in trichopteran family Hydropsychidae, 1 species in family Limnephilidae [70], and 1 species in family Leptoceridae [71], and are listed in Table 2.

For each caddisfly species, DNA was extracted from abdominal tissues from each specimen using the DNEasy Blood and Tissue kit (Qiagen, Düsseldorf, Germany) following the standard animal tissue extraction protocol with modifications as previously described [58]. Tissue was ground up in 180 µL of tissue lysis buffer ATL (Qiagen) using a mortar and pestle followed by 20 µL of protein kinase K (Qiagen, 600 mU/mL) which was added to the mixture and then incubated in a 55 °C water bath for 1 hour. Using the standard instrument protocol for purification of total DNA from animal tissue [59], the samples were processed on a QiaCube extraction robot (Qiagen) to complete the DNA extraction procedure. Extracted DNA was evaluated for yield and quality on a NanoDrop 2000 spectrophotometer (Thermo Scientific, Wilmington, Delaware, USA) and a Qubit 2.0 fluorometer (Life Technologies, Carlsbad, California, USA). DNA was stored in Eppendorf tubes (Eppendorf, Hamburg, Germany) at −20 °C until required [44].

The morphology-based identification of each species was further examined by *cytochrome c oxidase I* DNA barcoding. Polymerase chain reaction (PCR) products for the *COI* barcode sequence were obtained and sequenced for each specimen using standard methods [44],[72]. Sequences for each specimen were compared with reference sequences in the BOLD database [12], and in all cases yielded a species diagnosis consistent with that previously determined from morphological characteristics (data not shown).

### 2.2. Sequence preparation, assembly, and annotation

DNA libraries were prepared and samples were sequenced at the Next Generation Sequencing (NGS) Platform facility at the Children's Hospital Research Institute of Manitoba (Winnipeg, Manitoba, Canada). The DNA sample was sheared by sonication with an S220 Focused-Ultrasonicator (Covaris, Woburn, Massachusetts, USA). Fragment sizes were evaluated using a High Sensitivity DNA chip for the Bioanalyzer 2100 electrophoresis system (Agilent, Santa Clara, California, USA) using the standard manufacturer protocol. A TruSeq library preparation kit (NEB) was used to prepare an indexed library from each sheared sample for loading onto a MiSeq NextGen Sequencing Instrument equipped with either a MiSeq reagent V3 75X2 paired end reagent kit (6 samples) or a V3 300X2 paired end reagent kit (2 samples) (Illumina, San Diego, California, USA). In both cases, the specimens included in this study were processed simultaneously on the instrument with several other indexed libraries that will be described separately in future work. The sequences for each of the species included in this study represents about 10% of the data generated from a run of the MiSeq instrument.

The assembly process for *Anabolia bimaculata* (Trichoptera: Limnephilidae) and for *Triaenodes tardus* (Trichoptera: Leptoceridae) has already been described [70],[71]. For trichopteran species in family Hydropsychidae, the sequence reads for each species were assembled to the full mitochondrial genome reference sequence (GenBank voucher MF680449) and the complete ribosomal RNA repeat (GenBank voucher MF680448) from *A. bimaculata* [70] using Geneious version 10.1.2 [73]. In each case, MiSeq reads were mapped to the voucher sequence in 25 iterations at the “Medium-Low Sensistivity/Fast” setting of Geneious. In rare cases where the assembly produced large gaps (>30 bp), 1 kb of the consensus sequence of the assembly on each side of the gap were used as reference sequences for mapping the MiSeq reads in 5 iterations at the “Medium-Low Sensistivity/Fast” setting of Geneious. The resulting assemblies from both sides of the gap were then aligned with each other and with the original assembly that was mapped to *A. bimaculata* to produce a continuous sequence for the mitochondrial genome and the *rRNA* repeat for each species.

Sequences were annotated in Geneious. Secondary RNA structures were analyzed using the default settings of RNAstructure [74] and Mfold [75] software. Annotation of mitochondrial genes was facilitated by comparison with the mitochondrial genome sequences of *A. bimaculata* and *Eubasilissa regina* (Trichoptera: Phryganeinae, Genbank voucher NC_023374 [76]). Annotation of nuclear *rRNA* repeats was facilitated by comparison with *rRNA* repeat reference sequences from *A. bimaculata*, *T. tardus* (Genbank voucher MG201853 [71]), *Meroptera pravella* (Lepidoptera: Pyralidae, Genbank voucher MF073208 [69]), and *Samia cynthia ricini* (Lepidoptera: Saturniidae, Genbank voucher AF463459 [77]).

### 2.3. Phylogenetic analysis

Mitochondrial genome sequences from specimens collected at the Living Prairie Museum (Genbank Vouchers MF680449, MG201852, MG669121-MG669126) were combined with Trichopteran mitochondrial genome sequences from other geographic regions obtained from Genbank (Vouchers AB971912, KF756944, KP455290, KP455291, KT876876, NC 023374) previously published by other research groups [76],[78],[79]. These full-length mitochondrial genome sequences were then aligned with lepidopteran outgroups *M. pravella* and *S. cynthia ricini* (Genbank vouchers NC 017869, MF073207) [69],[77] in CLUSTAL Omega [80]. The nuclear *rRNA* repeats from each of the Living Prairie Museum Trichoptera (Genbank Vouchers MF680448, MG201853, MG669127-MG669132) were aligned with only the repeats from Lepidoptera outgroups *M. pravella* and *S. cynthia ricini* (Genbank vouchers MF073208, AF463459) [69],[81] because these are the first complete Trichopteran *rRNA* repeats to be reported.

The aligned mitochondrial genome and nuclear rRNA repeat sequences were each analyzed using the parsimony and maximum likelihood heuristic and bootstrap search algorithms implemented in PAUP* version 4.0b8/4.0d78 using default settings unless otherwise specified [82]. The best model for maximum likelihood phylogenetic analysis of both datasets were identified using jModeltest 2.1.7 [83] and likelihood ratio tests [84] and were determined in both cases to be the GTR + I + G (General Time Reversible) model (mitochondrial genomes: I = 0.1770, G = 1.0130, *rRNA* repeats: I = 0.3260, G = 0.6360). Parsimony and maximum likelihood (GTR + I + G) heuristic searches were carried out on the 658 bp barcode region of *COI* within the mitochondrial genome, the complete mitochondrial genome, and the complete nuclear *rRNA* repeat. Searches of each dataset were conducted using the following settings: 1 million maximum search replicates with random sequence addition, tree bisection and reconnection branch swapping on only the best trees, multiple trees saved at each step, and retention of the best trees. The bootstrap searches were conducted using the following settings: 1 million random sequence addition fast addition search replicates and retention of all groups compatible with 50% bootstrap consensus.

## 3. Results

### 3.1. Mitochondrial genome and nuclear rRNA repeat assemblies

Mitochondrial genomes and nuclear *rRNA* repeats were assembled for six caddisfly species in family Hydropsychidae and one species each in families Limnephilidae [70] and Leptoceridae [71]. Assemblies of the mitochondrial genome sequences ranged from 14,963 bp (*Trianodes tardus*, family Leptoceridae) to 15,185 bp (*Hydropsyche orris*, family Hydropsychidae) (Table 2). Most of the variation in sequence length in the mitochondrial genome between caddisfly species was in the control region, a noncoding sequence that services as an origin of replication for the mitochondrial genome and that is responsible for regulating transcription of mitochondrial genes [20]. The same gene order and arrangement was found in all of the mitochondrial genomes assembled in this study. It is the same as the ancestral mitochondrial gene order for all insects [76] and has been found in all other caddisfly species sequenced to date except for *Hydropsyche pellucidula* [71],[78].

Nuclear *rRNA* repeat assemblies varied in size from 7791 bp (*Cheumatopsyche analis*, family Hydropsychidae) to 9400 bp (*Anabolia bimaculata*, family Limnephilidae). Most of the variation in sequence length was in the 5′ external transcribed spacer and the 5′ non-transcribed spacer regions of the *rRNA* repeat [81]. Also present was some sequence length variation in ITS2, the most variable internal region of the repeat [85]. The gene order observed in the caddisfly *rRNA* repeats was identical in all species considered here and identical to that observed in most eukaryotic organisms [3].

### 3.2. Phylogenetic analyses

**Figure 1.**
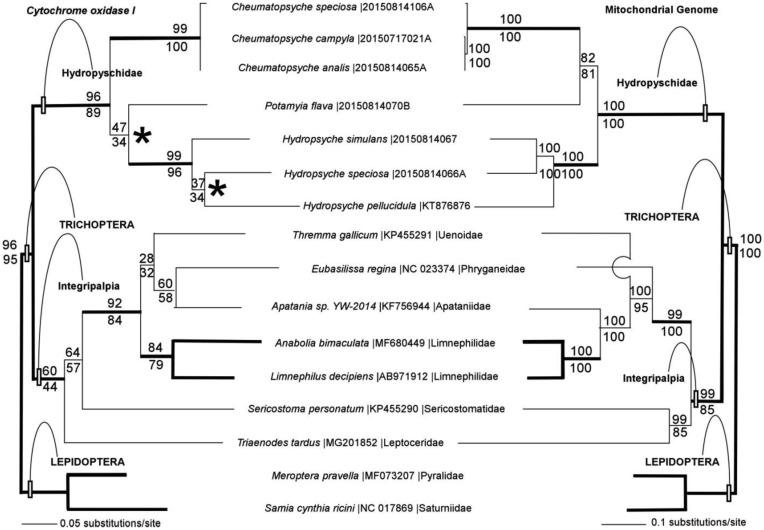
Phylogenetic tress reconstructed from *COI* barcodes (left) and complete mitochondrial genomes (right) using maximum likelihood and parsimony. Asterisks indicate where some of the four most parsimonious *COI* trees differ from the tree topology shown here. Portions of the phylogenetic tree that are congruent between the analyses of the *COI* and the mitochondrial genome datasets are indicated by bold lines on the tree. Maximum likelihood bootstrap values are shown above each node, parsimony bootstrap values are shown below the node.

Phylogenetic analysis of the *COI* barcode dataset produced four most parsimonious trees (length 1020 steps), one of which was identical to the single maximum likelihood tree (likelihood score 5040.9131) produced by this dataset (Figure 1). The most parsimonious trees differed from one another in the relationship of *Hydropsyche* species to one another, and in the placement of genus *Potamyia* relative to the genera *Hydropsyche* and *Cheumatopsyche*. In all cases, analysis of barcode sequences resulted in unresolved relationships between species in genus *Cheumatopsyche*. Strong maximum likelihood and parsimony bootstrap support was observed for the monophyly of insect order Trichoptera, for family Hydropsychidae, and for genera *Hydropsyche* and *Cheumatopsyche.* Except for moderate bootstrap support for family Limnephilidae, all of the other relationships between taxa had only weak bootstrap support from the *COI* barcode dataset.

Phylogenetic analysis of the mitochondrial genome dataset produced a single most parsimonious tree (length 31583 steps) with the same topology as the single maximum likelihood tree (likelihood score 158974.51910) (Figure 1). Species relationships in genera *Hydropsyche* and *Cheumatopsyche* were fully resolved by phylogenetic analysis of the mitochondrial genome. Bootstrap support was robust throughout, except for the node supporting the sister-clade relationship between *Potamyia* and *Cheumatopsyche*, where bootstrap analysis was more modest.

Monophyly of the Trichoptera, the Integripalpia, families Hydropsychidae and Limnephilidae, and genera *Hydropsyche* and *Cheumatopsyche* were supported by both *COI* barcode and mitochondrial genome datasets (bold branches on the phylogenetic trees, Figure 1). However, virtually all of the remaining relationships among taxa differ substantially between the trees generated from these 2 datasets derived from the mitochondrion.

**Figure 2.**
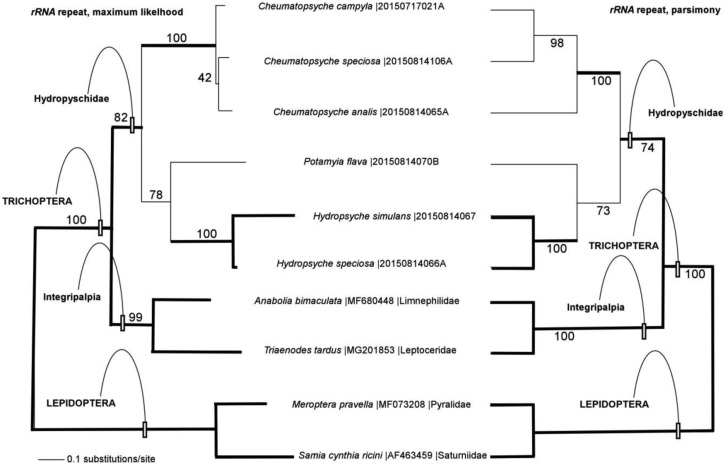
Phylogenetic tress reconstructed from nuclear *rRNA* repeats using maximum likelihood (left) and parsimony (right) methods. Portions of the phylogenetic tree that are congruent between the analyses of the nuclear *rRNA* repeat and mitochondrial genome datasets are indicated by bold lines on the trees. Maximum likelihood bootstrap values are shown above each node, parsimony bootstrap values are shown below the node.

Phylogenetic analysis of the nuclear *rRNA* repeat dataset produced trees with very similar topologies with as the result of parsimony (length 13211 steps) and maximum likelihood (score 65074.87230) searches, but the two methods reconstructed different relationships within genus *Cheumatopsyche* (Figure 2). Bootstrap support was very strong at most nodes, except for monophyly of the Hydropsychidae, the sister-relationship between genera *Potamyia* and *Hydropsyche*, and relationships within *Cheumatopsyche* (maximum likelihood only). In most cases, the *rRNA* repeat-based analyses also produced relationships among taxa that were the same as was found in phylogenetic analysis of the mitochondrial genome (bold branches on the phylogenetic trees, Figure 2) including the monophyly of order Trichoptera, the Integripalpia, families Hydropsychidae, and genera *Hydropsyche* and *Cheumatopsyche.* Exceptions to the congruency between mitochondrial genome-based and *rRNA* repeat-based trees are the relationships within genus *Cheumatopsyche* and the relationship of genus *Potamyia* to the genera *Hydropsyche* and *Cheumatopsyche.*

## 4. Discussion

### 4.1. Caddisfly mitochondrial genome structure

Assembled mitochondrial genomes and nuclear *rRNA* repeats can easily be recovered from shallow next generation sequencing of total cellular DNA (sometimes called genome skimming) due to their repetitive nature, as has been found in prior studies [49]–[51],[86]. The gene order and overall structure of caddisfly mitochondrial genomes is very consistent both among newly assembled sequences presented here and among previously reported mitochondrial genome sequences [70],[71],[76],[79]. The only exception to this general pattern is the sequence reported from *Hydropsyche pellucidula* (Hydropsychidae) that differs from other Trichopteran mitochondrial genomes in size (25 kb versus the typical ∼15 kb), in the arrangement of the mitochondrial rRNA genes (the *12S rRNA* was translocated from its usual position between the *16S rRNA* and the control region to a position between *cytochrome b* and *nad1*), in the atypical locations of *tRNA-P* and *tRNA-I*, and in topology (with a possibly linear mitochondrial genome structure) [78].

The mitochondrial genome sequences of *H. orris* and *H. simulans* share none of these features, suggesting that the reported rearrangements of the mitochondrial genome in *H. pellucidula* are probably either of very recent origin (occurring since the diversification of *Hydropsyche*) or may be attributable to experimental artifact. The *H. pellucidula* mitochondrial genome sequence is from an experiment involving the next generation sequencing of a metagenomic library containing multiple taxa followed by de novo assembly [78] so it is possible that contaminating sequences may have inadvertently been incorporated into the published sequence. It may be worthwhile to resequence and/or reassemble the *H. pellucidula* mitochondrial genome in order to verify the existence and timing of the reported rearrangements, since these features of the mitochondrial genome are often very useful for understanding relationships among insect taxonomic groups [87]. In any case, because the unique features of the reported *H. pellucidula* mitochondrial genome are autapomorphic, they are expected to have virtually no effect on the phylogenetic analyses performed in this study.

### 4.2. Performance of phylogenetic datasets

*COI* barcode-based phylogenetic analysis of the caddisfly species considered here produced multiple unresolved trees with poor bootstrap support both for species relationships within genera, and among the lineages representing different caddisfly families (Figure 1). The topology of the *COI* trees is also incongruent with relationships reported in previous analyses both between genera within family Hydropsychidae [88] and between trichopteran families [64],[65].

In contrast, phylogenetic analysis of complete mitochondrial genomes produces fully resolved trees with robust bootstrap support at nearly all nodes (Figure 1). With one exception, the mitochondrial genome-based phylogenetic relationships among the trichopteran families match those proposed previously on the basis of other data sets [64],[65]. The principal difference is that in this analysis, the Apantaniidae and the Limnephilidae are sister clades, with the Uenoidae as a near outgroup; while in prior analyses the Apantaniidae and Uenoidae were sister clades, with the Limnephilidae as the outgroup. This part of the trichopteran phylogenetic tree (which included several other families not sampled in the current study) was not fully-resolved by prior work based on smaller data sets [64], so it is not surprising that the larger number of informative characters found in the mitochondrial genomes introduces some changes in this portion of the topology.

Maximum likelihood and parsimony methods for phylogenetic reconstruction produced nearly identical tree topologies from the *rRNA* repeat dataset, except for the arrangement of species within the genus *Cheumatopsyche* (Figure 2). Neither of these topologies matches the arrangement of *Cheumatopsyche* species in the mitochondrial genome-based tree (or the arrangement in the *COI* barcode tree where the basal *Cheumatopsyche* node is unresolved) (Figure 1). There are several factors that might contribute to these patterns of phylogenetic discordance among these very closely related (and in all probability, recently diverged) species, including retained ancestral polymorphisms, lateral transfer of mitochondria between lineages, and possibly selection on the mitochondrial genome and/or the nuclear *rRNA* repeat [89]. Other than the relationships within *Cheumatopsyche*, the remainder of the nuclear *rRNA* repeat-based trees is topologically similar to the mitochondrial genome-based tree, with one exception. In the *rRNA* repeat tree, *Potamyia* is sister to genus *Hydropsyche*, in agreement with the weakly supported arrangement from the *COI* barcode tree, but incongruent with the strongly supported sister relationship between *Potamyia* and *Cheumatopsyche* from the mitochondrial genome data set in this study (Figure 1) and by a prior study [88]. Bootstrap analysis of the *rRNA* dataset shows robust bootstrap support at most nodes, except for some of the nodes that differ between this data set and the mitochondrial genome-based dataset. Better taxon sampling of additional genera in the Hydropsychidae as well as additional families in the Trichoptera will help to break up the longer branches of the phylogenetic tree may aid in improving the robustness of the reconstructions for some of these nodes in the *rRNA* repeat trees [90].

### 4.3. Building a better barcode

Investigators have widely different perspectives on the value of conventional DNA barcoding strategies, much of which can be traced to whether these strategies are appropriate for answering the questions being addressed within their research programs [14]. Researchers whose primary interest is to identify unknown specimens, to associate morphologically disparate individuals (due to life stage, sexual dimorphism, or other kinds of variation) from the same species, or to quantify individuals of a given mitochondrial haplotype in the environment might be completely adequately served by the short barcode sequences (including the *COI* fragment) currently in use [29],[63],[91], at least in some taxonomic groups [13]. Others with research questions that require inferences about the relationships among organisms often find that the information content of *COI* barcodes is insufficient for their purposes [18],[37],[53]. However, even subjects such as species delimitation [92],[93], cryptic species identification [21],[37], or hybridization and organelle capture [16],[41],[94], research topics for which DNA barcoding is supposedly highly suitable [14], cannot be demonstrated without reference to sequences from the nuclear genome, examination of morphological or other phenotypic traits, or both [13],[95]. Simply stated, the limited information content of conventional plastid-based barcodes due to their small size, and their propensity for lateral transfer between lineages due to their location within the mitochondrial genome means that they cannot be used to effectively address certain research questions [13],[14],[18],[35],[37],[53].

The desirability of a standard set of genetic markers that can be used to identify nearly any organism is clear, and if the phylogenetic species concept is to be invoked in order to operationalize species identification, the markers used should possess qualities that will allow them to be phylogenetically informative. The short barcode sequences currently in use were developed in the context of experimental technologies that placed constraints on the size and location of the barcode regions that are no longer universally applicable (Table 1). While some have argued that next generation sequencing may make DNA barcoding obsolete (e.g., [13],[53]), I am more convinced by the argument that these new sequencing technologies may revolutionize DNA barcoding [96], because, akin to the solution of the Y2K problem, they allow the expansion of sequence identifiers so as to increase their information content. In particular, by using next generation sequencing to expand the barcode sequence regions to encompass entire plastid genomes by genome skimming, this greatly increases our ability to distinguish between species that have recently diverged from one another by sampling more sites that might have undergone mutation [44]. Similarly, plastid genomes include genes that evolve at dramatically different rates [13],[72], and by sampling and sequencing them in their entirety it becomes possible to resolve deeper phylogenetic nodes with robust bootstrap support that are unresolved in conventional barcode-based trees (Figure 1) [18]. Even better, because mitochondrial genomes contain the *COI* sequence and chloroplast genomes include the *rbcL* and *matK* genes, they are “backwards compatible” with the conventional barcodes currently in use.

If combinatorial analyses involving sequences from more than one source are included within the definition of barcoding (as already in use for plant barcoding [6]), one can further expand the sources of DNA barcode information to include the nuclear *rRNA* repeat which in turn includes the *ITS2* region used for DNA barcoding in fungi [3] and the *28S rRNA*, the eukaryotic homologue of the *16S rRNA* used for barcoding in bacteria [4],[5]. Unlike plastid genomes, the nuclear *rRNA* repeat is bi-parentally inherited as a component of the nuclear genome [97], and thus may not be as prone to phylogenetic distortions due to lateral transfer and organelle capture. Even when lateral transfer is unlikely, the phylogenetic hypotheses produced by organelle genomes and the *rRNA* repeat may not always be congruent, (as is the case between the genera *Cheumatopsyche*, *Potamyia*, and *Hydropsyche*, in this study, see Figures 1 and 2), but each of these genera are clearly distinguished by both data sets, and this may set the stage for further work examining additional genetic regions if the relationships of these genera are of particular importance for addressing a given research question. In plants, where chloroplast genomes, mitochondrial genomes, and nuclear *rRNA* repeats can be recovered from genome skimming relatively easily [48],[51] it may be possible for researchers to “triangulate” and use the phylogenetic signal from all 3 sources to identify specimens and reconstruct the evolutionary history of a group.

What I am proposing is by no means the only way to employ next generation sequencing methods for phylogenetic reconstruction, but it does have several advantages over some of the available alternatives. For example, a recently announced set of PCR primers for amplifying 30 nuclear genes in the Lepidoptera, comprising some 11 kb of DNA sequence, is based entirely on slowly evolving coding sequences [98], potentially making them valuable for resolving interfamilial relationships, but likely with limited applications for more recent species divergences. It is also unknown to what degree the primers for these nuclear regions will work in taxa outside of the Lepidoptera and there are few comparable sequences in the databases to compare with data generated from these primers. Conversely, restriction site associated DNA sequencing (RADSeq) can be used to generate extremely large phylogenetic data sets that are often quite valuable for resolving relationships among closely related species [40]. However, as more taxa and especially more distantly related data are included in the analysis, the proportion of missing data increases and the proportion of informative characters often decrease in RADSeq data sets. Thus, both of these approaches lack features of standardization that are present in next generation barcodes, making them less attractive for answering certain kinds of research questions.

More compatible with next generation barcodes are mitogenomic approaches which enrich target sequences by PCR [52],[54],[99] and target enrichment approaches which use anchored probes to pull target sequences out of genomic pools, followed by sequencing [100]. If probes are designed to match (or primers are designed to amplify) entire mitochondrial genomes, entire chloroplast genomes, and complete nuclear *rRNA* repeats, these approaches may allow larger numbers of species to be included within a single lane or run of a next generation sequencer. Target enrichment by anchored probes may be a particularly effective method for obtaining the sequences of these regions from rare species preserved as specimens in museum collections and may ultimately also be a cost-effective method for extracting fragments from these genetic regions from recently collected specimens as well. The opportunity to deploy next generation barcodes to answer research questions using all of these methods is expected to continue to increase as next generation sequencers become more available and as the cost of running the instruments continues to drop.

## 5. Conclusion

While several other authors have suggested that next generation sequencing will have profound effects on how barcoding is conducted [13],[26],[53],[96], this is the first explicit proposal that genome skimming by next generation shotgun sequencing be used to enlarge conventional DNA barcode identifiers and upgrade current barcoding strategies. This expansion increases the information content of barcodes, giving them properties that are appropriate for the statistical operations used in phylogenetic analysis. This is analogous to expanding the year identifiers to resolve the Y2K problem in computer science so that they have a sufficient number of digits to permit mathematical operations and chronological sorting [10]. The improved functionality of the proposed next generation barcodes over conventional *COI* barcodes was demonstrated by comparing their effectiveness in reconstructing the phylogenetic history of the Trichoptera.
